# The clonal heterogeneity of colon cancer with liver metastases

**DOI:** 10.1007/s00535-023-01989-6

**Published:** 2023-04-12

**Authors:** Guanxuan Chen, Wanqi Zhu, Yang Liu, Liwen Zhang, Li Xie, Xingguo Song, Xianrang Song

**Affiliations:** 1 Department of Intensive Care Unit, Shandong Cancer Hospital and Institute, Shandong First Medical University and Shandong Academy of Medical Sciences, Jinan, Shandong People’s Republic of China; 2 Department of Research and Education, Shandong Cancer Hospital and Institute, Shandong First Medical University and Shandong Academy of Medical Sciences, Jinan, Shandong People’s Republic of China; 3 Shanghai OrigiMed Co., Ltd, Shanghai, People’s Republic of China; 4 Department of Clinical Laboratory, Shandong Cancer Hospital and Institute, Shandong First Medical University and Shandong Academy of Medical Sciences, Jinan, Shandong People’s Republic of China; 5 Shandong Provincial Key Laboratory of Radiation Oncology, Shandong Cancer Hospital and Institute, Shandong First Medical University and Shandong Academy of Medical Sciences, Jinan, Shandong People’s Republic of China

**Keywords:** Clonal heterogeneity models, Colon cancer with liver metastases (CCLM), Subclones, Whole-exome DNA sequencing

## Abstract

**Background:**

Colon cancer with liver metastases (CCLM) characterized by genetic heterogeneity is an evolutionary process leading to variations in response to selective pressure, but the underlying evolutionary models still remains unclear.

**Methods:**

Total of 30 samples, including primary tumor and two to four matched liver metastases from 8 treatment-naïve patients with CCLM were collected, and subjected to whole-exome DNA sequencing. PyClone was used to calculate intra and inter-tumor heterogeneity, LICHeE was used to reconstruct the cancer phylogeny trees and investigate the subclonal composition.

**Results:**

The genetic differences were observed between primary and metastatic lesions, as well as among multiple metastases in all patients. The natural history models of colorectal cancer in each case were identified, including parallel, linear, and branching evolution. Liver metastases could originate from primary lesions or other metastases. Pathway and process enrichment analysis also showed obvious heterogeneity and enhancement of several molecular functions.

**Conclusions:**

Our data reveal the genetic and heterogeneity between primary and metastatic lesions, as well as among multiple metastases and provide genomic evidence for clonal heterogeneity for CCLM.

**Supplementary Information:**

The online version contains supplementary material available at 10.1007/s00535-023-01989-6.

## Introduction

Despite incessant improvement of surgery, radiotherapy and chemotherapy, colon cancer (CC) still is one of the most common causes of fatalities in both men and women worldwide [1]. Distant metastasis is a lethal consequence of cancer progression in CC patients, liver is the main metastatic site. Recent investigations have highlighted CC with liver metastasis (CCLM) is a genetic evolutionary process characterized by genetic heterogeneity, leading to variations in response to selective pressure and treatment outcome [2–5].

It is widely appreciated that the majority of CRC develop as a consequence of the progressive accumulation of genetic alterations in evolving clones of tumor cells, which also plays an essential role in the metastatic process of CRC [6]. Thus, modeled mCRC evolution may provide the evolution-based categorization of genomic alterations and the inferred mutational landscape. The natural history of tumor progression follows four evolutionary pathways: parallel evolution displaying similar genotypic and phenotypic modifications under similar environments, sequentially acquired driver mutations over time called linear evolution, genetically distinct with many standing lineages supporting the branch evolution, mutations in genes fortuitously relapses undergoing neutral evolution [7, 8]. In addition, studies find that the cellular heterogeneity and the constant evolution of CRC complicates the design of effective treatment regimens [9]. The influence of genetic heterogeneity and evolution on the response to neoadjuvant treatment has been analyzed, the results revealed substantial changes in subclones, and selective modification and subclonal populations were enriched after treatment [10].

The evolutionary process of tumor generates subpopulation cells termed subclones, which share subsets of mutations, their divergence result in genetic and molecular heterogeneity [2, 3]. Due to selective pressure, mutationally distinct subclones were selected by advantage; each of those subclones possesses a characteristic clinical property (e.g., drug sensitivity, growth rate, or metastatic potential) [11]. For CCLM, a range of subclones are competing and positively selected according to their ability to metastasize and adapt to the liver environment. Thus, subclonal cluster analysis may shed light on the characteristics of molecular biology of CCLM [2, 12–14]. Hence, understanding the clonal composition and phylogenetic tree may help us understand the biology and evolution of CCLM, as well as guide the design of combinatorial therapies. Nevertheless, the heterogeneity and evolution of CCLM remains unknown.

In the present study, we retrospectively collected matched tissues of primary and multiple liver metastases from treatment-naïve patients with CCLM. Subsequently, we performed genomic profiling of 30 samples (i.e., eight primary tumors and 22 CCLM samples) from eight patients. The objective of this study was to describe the genetic heterogeneity, reconstruct the cancer evolution history and analyze the function of subclones.

## Materials and methods

### Patients

Thirty samples including matched 8 primary and 22 CCLM samples were collected from 8 CC patients admitted to Shandong Cancer Hospital affiliated to Shandong First Medical University and Shandong Academy of Medical Sciences from January 2013 to November 2019. The CCLM patients were diagnosed according to the tumor specimens’ histological examination and the 8th Edition of the AJCC Staging System. All patients didn’t receive any anti-tumor treatment before sampling. This study was approved by Ethics Committee of Shandong Cancer Hospital affiliated to Shandong First Medical University and Shandong Academy of Medical Sciences. Informed consent was obtained from all individuals.

### DNA extraction

Genomic DNA was extracted from tumor samples according to the following procedures. A 4-μm section of a hematoxylin and eosin–stained slide of a FFPE sample underwent a pathologist review to ensure each sample at least had the area of 1cm^2^, nucleated cellularity of 80% and tumor content of 20%. Ten unstained FFPE sections (total 40 μm) were used, generating 0.5–2 μg of DNA using the QIAGEN QIAamp DNA Mini Kit (QIAGEN) according to the manufacturer's instructions.

### Whole-exome sequencing

WES libraries were constructed using KAPA hyper prep kit and 50–500 ng of double stranded DNA was fragmented to ~ 250 bp by sonication. Subsequent library construction was done using KAPA Hyper Prep Kit (KAPA Biosystems) for end repair, d A addition and adapter ligation was performed, followed by PCR amplification and Qubit™ dsDNA HS Assay quantification (Thermo Fisher Scientific). Samples yielding < 40 ng of extracted DNA or < 500 ng of pre-capture library were excluded for further sequencing. The SureSelect Human All Exon V6 from Agilent was used to target all exons. Hybridization capture followed the manufacture’s protocol Hybridization capture of DNA libraries using xGen® Lockdown® Probes and Reagents (Integrated DNA Technologies, Ver.4). Post-capture libraries were mixed together, denatured and diluted to 1.01 ~ 1.1 nM and subsequently sequenced on Novaseq 6000 with paired-end reads 2 × 150 bp by following the manufacturer’s protocols. (Illumina, Inc.)

### Data analysis

WES sequencing reads after exclusion of low-quality reads were mapped to the UCSC hg19 reference sequence with BWA (version 0.7.15, http://bio-bwa.sourceforge.net/). PCR duplicates were removed by Picard (version 2.0.1, http://broadinstitute.github.io/picard/), and recalibrated by the Base Recalibrator tool from GATK (version 4.0.6.0, https://software.broadinstitute.org/gatk/). Somatic variants were detected using Mutect (version 2) on exome data of tumor. Annotation of variants was performed by Annovar (version 2017/07/17) [15], on Refseq gene models (version 2017/06/01). Known germline variants were filtered from dbSNP (version 147), database of the 1000 Genomes, NHLBI Exome Sequencing Project (ESP6500), Exome Aggregation Consortium (EXAC), and Genome Aggregation Database (gnomAD). A stringent downstream filter comprised of the following criteria was used to obtain high quality somatic variants: a minimum of 30X coverage; Variant Allele Fraction (VAF) >  = 1% and at least 5 variant supporting reads in the tumor sample, strand bias <  = 0.95; after that, mutations in the non-coding regions (3’UTR, 5’UTR, Intron, gene intergenic etc.) were removed.

For each tumor, SCNAs were inferred by CNVkit (version 0.9.5, https://cnvkit.readthedocs.io/en/stable/pipeline.html) using Circular Binary Segmentation algorithm with default parameters. Segment-level ratios were calculated and log2 transformed. A log2 ratio cutoff of ± 0.8 was used to define SCNA amplification and deletion [16]. As there’s no matched normal could be obtained, we used pooled normal as control to detect CNV [17]. Gene rearrangements different chromosomes and long indels located in the same chromosome were analyzed by pipelines published in 2019 by OrigiMed lnc. [18]. Mutation landscape was visualized by R package maftools. TMB was defined as the number of somatic mutations (including base substitutions and indels) in the coding region. To reduce sampling noise, synonymous alterations were also counted [19]. To calculate the TMB, the total number of mutations counted was divided by the size of the coding sequence region of the Agilent SureSelect Human All Exon V6. HLA-I genotyping was assessed by WES and patients were considered fully heterozygous at HLA-I if they had six different HLA-I alleles [20]. To uncover the immune-associated gene profile of primary and metastases, all immunologically relevant genes were extracted from the ImmPort (https://www.ImmPort.org/).

Heterogeneity analysis was performed with PyClone (version 0.13.0, default parameter) [21]. For inputs to PyClone, reference, variant read depths and copy number at each locus was taken from the output of previous analysis. PyClone was run with 10,000 iterations and a burn-in of 1000, as suggested by the authors.

### Reconstruction of clonal evolution

For construction of the phylogenetic tree, multi-sample tumor phylogenies and tumor subclone decomposition were analyzed using LICHeE (Lineage Inference for Cancer Heterogeneity and Evolution) [22]. LICHeE is a method that use SSNVs from multiple samples of individual cancer patient to reconstruct cancer cell lineages and the evolutionary network to illustrate evolutionary timing relationships between each cluster pair, where each node corresponds to an SSNV cluster (the root represents the germ line) and each edge between two nodes, denotes that parent node could be an evolutionary predecessor of child node (SSNVs in parent cluster could have ‘happened before’ SSNVs in child cluster). The LICHeE algorithm was implemented in Java. It is the open source and freely available online (LICHeE Github Repository. http://viq854.github.io/lichee/).

### Pathway analysis and statistical analysis

The pathway/ontology enrichment analysis of mutations genes was performed using the Metascape (http://metascape.org). The statistical tests were conducted at a two-sided level of significance of 0.05 in R environment. Immunohistochemical analysis for Ki67 and cleaved-caspase-3 was performed using paraffin sections. Immunohistochemical staining was carried out for cleaved-caspase-3 (1:200; Servicebio, GB11009-1) and Ki67 (1:600; Servicebio, GB121141).

## Results

### Workflow

**Fig. 1:**
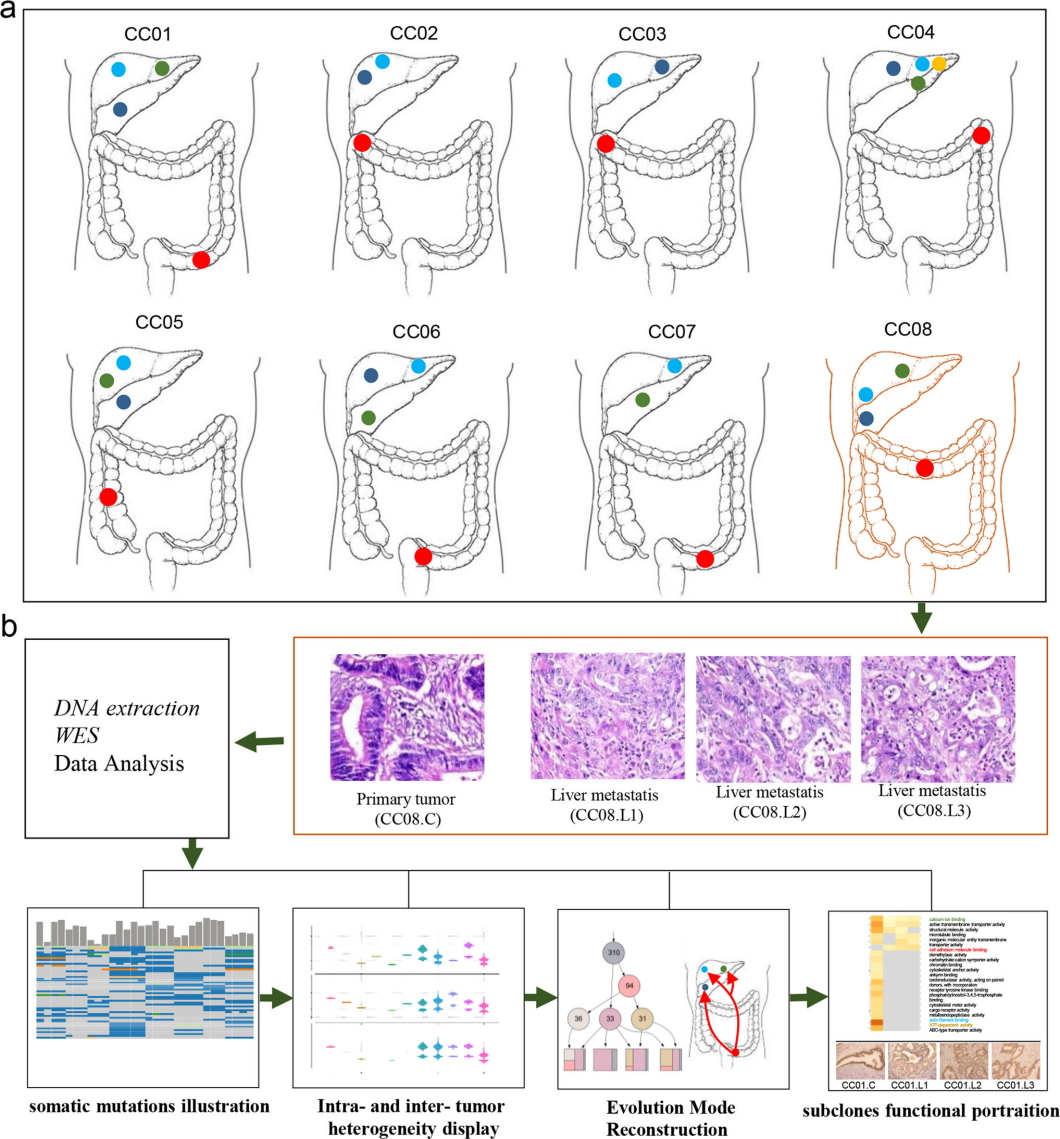
Overview of patient cohort, samples, and study design. **a** Schematic diagram of the primary and paired multiple liver metastases locations for all eight patients. **b** Identification of pathology of colon primary and matched multiple metastasized liver tumor tissues, sequencing and clonal evolution analysis

In current study, we sequenced 30 unique bulk tumor samples (matched 8 primary tumor samples and 22 liver metastatic samples) from 8 patients with CCLM, the primary and metastatic tumors were resected at the same period and no patient received chemoradiotherapy pre-operation. All tumors were microsatellite stable, and other characteristics information about the patients and samples are provided in Table 1. The collected samples were sequenced using whole-exome sequencing, and the workflow was illustrated in Fig. 1: firstly, samples from each of the eight patients were shown (Fig. 1a), and the specific locations of the primary and metastasis loci were shown in Table 1. Next, the HE stain of the primary and multiple metastases were underwent review, followed by next-generation whole-exome DNA sequencing, and the CC08 HE stains were depicted in Fig. 1b as an example. Finally, a series of data analyses were completed, including somatic mutations illustration; intra- and inter-tumor heterogeneity illustration by PyClone, evolution mode reconstruction as well as biological function assay.

**Table 1:** Patients and samples characteristics

Patient ID	Gender	Age	TNM*	Stage*	Histopathology	MSI status	Pre-op CRT	Status	Survival time (days)	Sites	Sample ID
CC01	Male	59	pT4N2M1	IV	MD	MSS	No	Death	826	Sigmoid colon	CC01.C
										Right liver lobe	CC01.L1
										Liver	CC01.L2
										Liver	CC01.L3
CC02	Male	71	pT3N0M1	IV	MD	MSS	No	Alive	368	Hepatic flexure Liver Liver	CC02.C CC02.L1 CC02.L2
CC03	Female	56	pT4N0M1	IV	MD	MSS	No	Death	270	Hepatic flexure	CC03.C
										Right liver lobe	CC03.L1
										Left liver lobe	CC03.L2
CC04	Male	52	pT3N2M1	IV	MD	MSS	No	Alive	811	Splenic flexure	CC04.C
										Left liver lobe	CC04.L1
										Liver segment IV	CC04.L2
										Liver segment IV	CC04.L3
										Liver segment V	CC04.L4
CC05	Male	69	pT4N1M1	IV	MD	MSS	No	Death	1093	Asending colon	CC05.C
										Right liver lobe	CC05.L1
										Right liver lobe	CC05.L2
										Right liver lobe	CC05.L3
CC06	Male	43	pT4N1M1	IV	MD	MSS	No	Alive	960	Rectosigmoid junction	CC06.C
										Liver segment IV	CC06.L1
										Liver segment VI	CC06.L2
										Right liver lobe	CC06.L3
CC07	Male	52	pT4N0M1	IV	MD	MSS	No	Death	2136	Sigmoid colon	CC07.C
										Liver segment IV	CC07.L1
										Liver segment V	CC07.L2
CC08	Female	56	pT3N1M1	IV	MD	MSS	No	Alive	2819	Transverse	CC08.C
										Liver segment IV	CC08.L1
										Liver segment V	CC08.L2
										Liver segment V	CC08.L3

^*^AJCC 9^th^ Edition

*Pre-op CRT* preoperative chemoradiotherapy, *MD* moderately differentiated

### Diversity between primary and metastases in treatment-naive CCLM

**Fig. 2:**
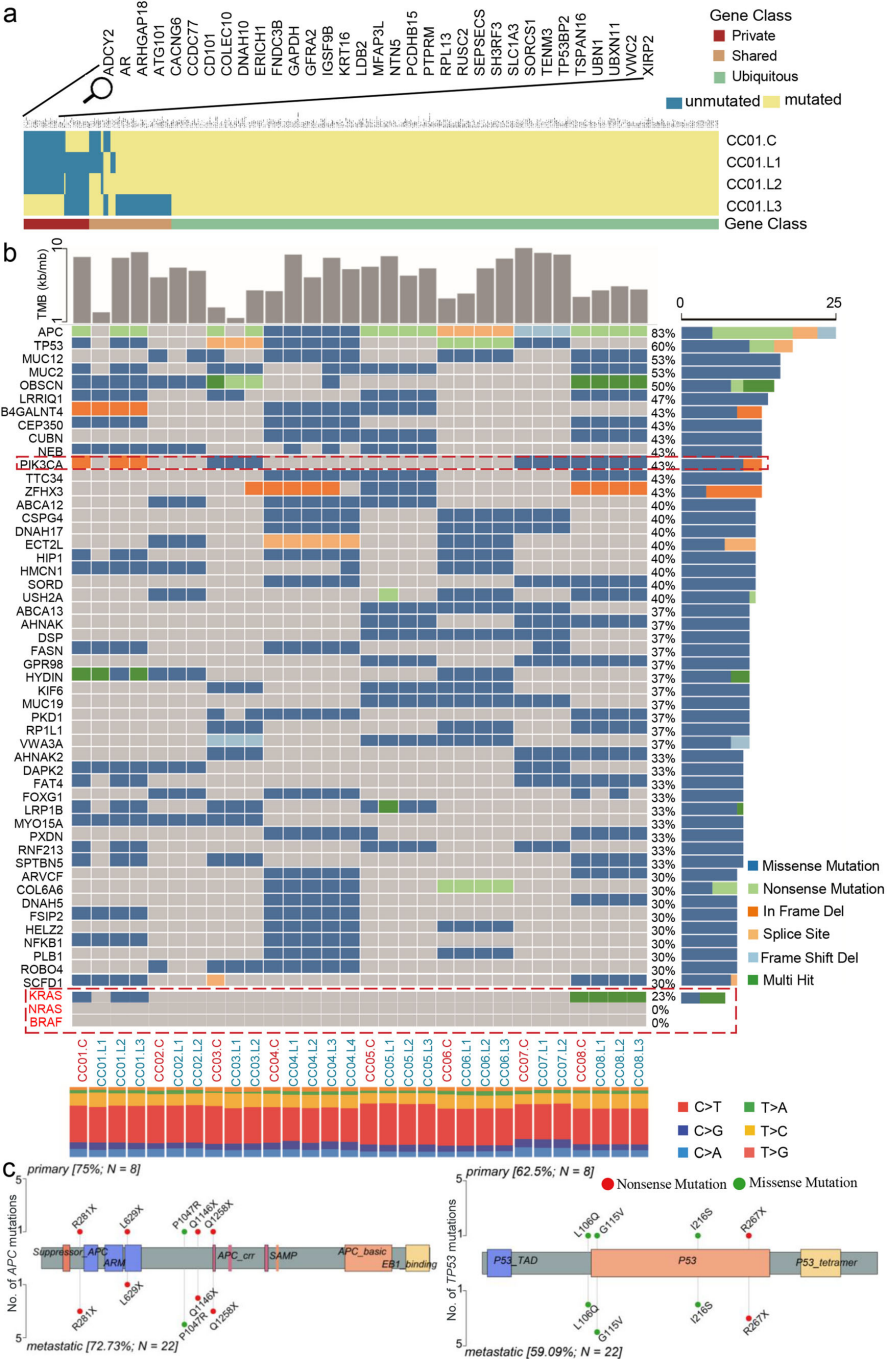
Comparison of somatic mutations in treatment-naïve colon cancer between primary and paired multiple metastases. **a** Heatmaps show the distribution of all nonsynonymous mutations. Presence (yellow) or absence (blue) of each mutation is marked for each sample within one individual CC01. Private is sample specific (red), shared means mutations occurring in more than one tissue (brown) and ubiquitous is mutations identified in each tumors (green). The mutated genes are listed in the supplementary materials. **b** Oncoplot showing the TMB in each sample (top panels); somatic mutation landscapes of the top 50 most frequently mutations (middle panels); the base mutation type distribution of each sample (bottom panels). In the middle panels, genes mutation frequency and mutation type are indicated on the right. **c** Lollipop mutation diagrams of primary (pointing up) and metastases (pointing down). Different color patches represent different domains, and y-axes means the number of mutations in primary and metastases, note that multiple mutation in a gene may occur from some case (color figure online)

An individual mutation profile was generated for each patient to present the genetic homogeneity and heterogeneity between primary and metastatic lesions (supplement materials S1). As shown in Fig. 2a and supplement materials S1, “Private” and “Shared” stood for heterogeneity, whereas “Ubiquitous” stood for homogeneity, evidently, mutations differed significantly between primary lesions and metastases, as well as among multiple metastases in all eight patients.

Moreover, the mutation information of the top 50 genes with the highest frequency, as well as the clinically important genes including *KRAS*, *NRAS*, *BRAF*, and *PIK3CA* were summarized and illuminated in Fig. 2b., to further illuminate the characteristics of mutated genes in each patient. The mutation frequency of *PIK3CA* was 43% (cases CC03, CC07, and CC08), two types of mutations were observed in which H1047R was dominant; whereas *KRAS* mutation was detected in only two patients (i.e., missense mutation in CC01 and multi-hit mutation in CC08). Notably, the mutation of either *PIK3CA* or *KRAS* in the primary lesion did not variate but was missing in metastatic foci (Table S1). Unexpectedly, no mutations of *NRAS* and *BRAF* were detected in all of the enrolled samples, which might attribute to the small patient sample size.

The type of alterations exhibited diversity between primary lesions and multiple metastases as evidence from the landscape. For instance, *OBSCN* mutations in case CC03, were multi-hit in primary but nonsense in metastases. The difference of tumor mutational burden (TMB) between samples is obvious in CC01, CC03, CC04, CC05 and CC06, the metastases had a higher TMB, but no significant difference was observed between primary and metastasis (Fig. S1a). Additionally, heterozygous HLA-I genotypes were detected in all of patients (30/30, 100%). Next, all immunologically relevant genes were extracted from the ImmPort. The mutations were picked out based on the immune-associated genes reflecting different statuses in the immune context of microenvironment (Fig. S2).

Finally, the mutation gene loci were diagramed for the top 10 most frequently mutated genes and the clinically important genes of *KRAS* and *PIK3CA*, which were depicted by lollipops (Fig. 2c and Fig. S1b). Subsequently, we found different mutation loci between the primary and metastases in *NEB*, *OBSCN* and *B4GALNT4*. These results indicate the intricate heterogeneity between primary and metastases, even when they shared the same mutation genes, the mutation form or loci varied greatly.

### Intra- and inter- tumor heterogeneity in treatment-naive CCLM

**Fig. 3:**
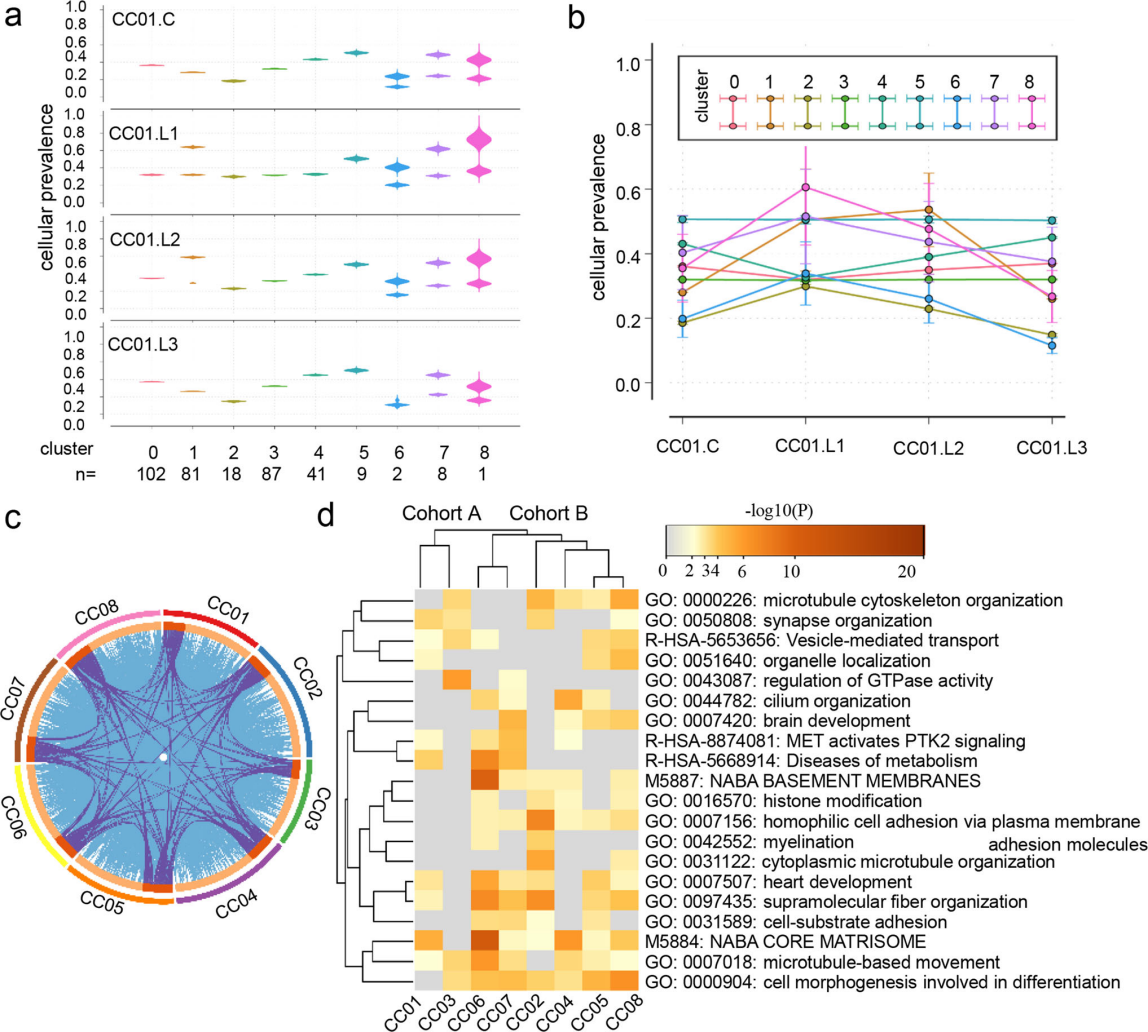
Intra and inter-tumor heterogeneity and subclones functional analysis. **A **The cellular prevalence of mutational clusters (vertical axis) in each sample is depicted at the left, width of the violin plots indicating the distribution of probabilities is generated by PyClone. The clusters order is along the x axis, and and n represents the amount of somatic single nucleotide variant included in that cluster. Composition profiles calculated from the PyClone model (right) plot the mean cellular prevalence of the variants in each cluster in each sample. Vertical lines at each point represent one standard deviation. **b** The Circos plot is performed by Metascape, it shows how genes from the clustered mutation gene lists overlap. On the outside, each arc represents the identity of each patient clustered mutation gene list. On the inside, each arc represent a gene list, where each gene has a spot on the arc. Dark orange color represents the genes that appear in multiple lists and light orange color represents genes that are unique to that gene list. Purple lines link the same gene that are shared by multiple gene lists. Blue lines link the different genes where they fall into the same ontology term (the term has to statistically significantly enriched and with size no larger than 100). **c** Enrichment analysis is performed by Metascape. The heatmap cells are colored by their *p*-values, light gray cells indicate the lack of enrichment for that term in the corresponding gene list (color figure online)

Intra-tumor and inter-tumor heterogeneity can act as a substrate for genetic diversity and tumor evolution. Pyclone, a software to predict subclone numbers and infer clonal structures, was employed to evaluate the heterogeneity of CCLM. Nine clusters were identified across all samples in case CC01, and different cluster included diverse mutations, for example, cluster 0 obtained 102 somatic mutations, and cluster 1 included another 81 mutation genes. The cellular prevalence displayed differently across the primary and matched multiple metastases, the variation trend was obvious in cluster 1 (mean 0.260–0.536), but seemed unchanged across samples in cluster 3 (mean 0.318–0.320) (Fig. 3a, b). Consistently, the similar phenomenon was also observed in the other seven patients (Fig. S3 a-b), indicating the intra- and inter-tumor heterogeneity.

Next, a circus plot was applied to demonstrate the relationship between the identified mutation genes in all clusters. As shown in Fig. 3c, although patients shared fewer genes with each other (purple links), they exhibited more functional overlaps (blue links). This finding implied that mutations might be involved in different biological processes. In addition, we subsequently conducted gene set enrichment analysis of biological functions for the inferred clusters. Accordingly, two cohorts were hierarchically clustered based on Kappa-statistical similarities among their gene memberships (Fig. 3d). We hypothesized that these two cohorts may be linked to different prognoses. Further study involving more cases is required to confirm our findings.

### Three different phylogenetic evolution model of CCLM

**Fig. 4:**
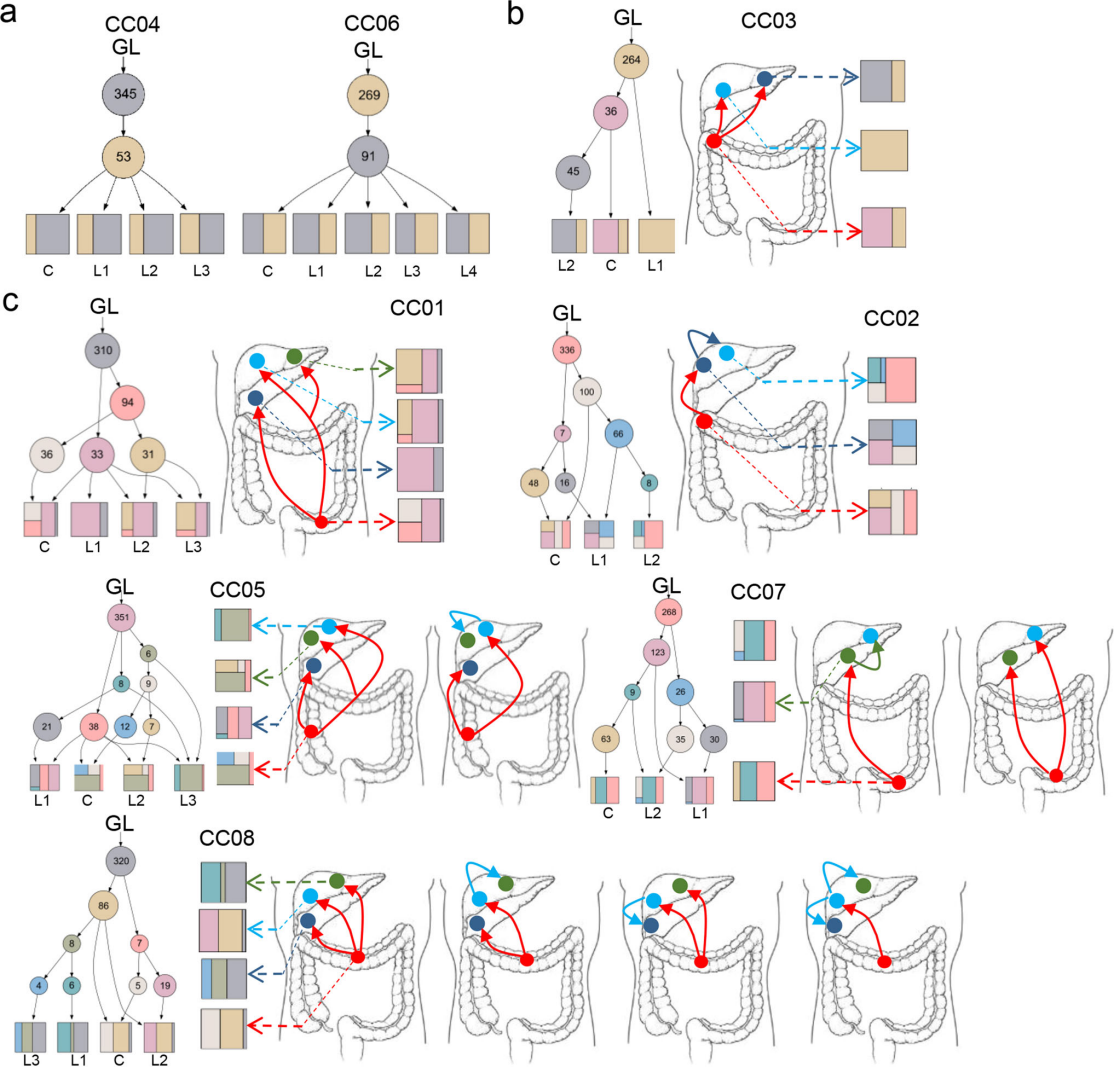
Tumor phylogenetic trees of colon cancer with multiple liver metastases. Phylogenetic trees inferred by LICHeE are shown for each patient (**a**, and left in **b**, **c**), showing each sample evolutionary process. The number in each node is the amount of somatic single nucleotide variant included in that subclone, the decomposition for each sample in the tree is displayed and how much each node contributes to the genomic makeup of the sample. Schematic outlines of tumor metastatic progression are shown on the right in B and C, with the clonal subpopulation of each sample shown. Arrows represent seeding clones between samples. Two possible scenarios are investigated in case CC05 and CC07, and four possibility in case CC08. GL, germ line. The asterisk stands for transition subclones

We also sought to elucidate the evolutionary process of CCLM. Therefore, the Lineage Inference for Cancer Heterogeneity and Evolution (LICHeE) method was used to analyze the evolutionary model of eight patients. Three different phylogenetic models were identified (i.e., parallel, linear, and branching evolution) (Fig. 4). As shown in Fig. 4a, sibling clades deriving from a single ancestral genotype were found in two cases (i.e., CC04 and CC06), indicating parallel evolution in primary lesions and different metastases. For cases CC04 and CC06, there were no novel mutations detected in the liver metastases; all mutations evolved from the primary lesions. Moreover, a linear evolution model was noted in case CC03. Both liver metastases lost 36 mutations compared with the primary lesion, whereas one metastasis gained 45 novel mutations (Fig. 4b left). According to the LICHeE algorithm, we concluded that L1 first metastasized from the primary lesion, followed by L2 (Fig. 4b right).

Another model revealed mixed lineages evolution in the rest cases, displaying diverse subclones in primary and metastases (Fig. 4c). Compared to primary sites, all the metastatic sites developed novel mutations to some extent, these mutations were formed during tumor progression, some of which acquired metastatic potential. For example, in case CC01, mutations were grouped into 5 subclones, in which subclone with 33 mutations was found in both primary and metastatic sites. During tumor progression, L2 and L3 first moved to liver after primary site developed subclone with 94 mutations, followed by developing shared 31 mutations respectively, while primary site developed other 36 mutations, and L1 transferred to liver.

Based on the same procedure, we speculated the evolutionary process from the phylogenetic trees in case CC02 (Fig. 4c). L2 was derived from L1 from subclone7, and evolved into a unique subclone 6 at the liver metastases, which is also consistent with a metastasis-seeding metastasis model. For cases CC05 and CC07, we investigated two possible scenarios according to the phylogenetic trees. For example, in case CC05, the L2 was derived from subclone 8, which could be acquired from the primary lesion or the L3 metastasis. There were four possibilities in case CC08: L1 and L3 could emerge from the primary lesion or the L2 metastasis in subclone 1.

Overall, these data indicated that the ancestral genotype may not be present in all samples. The proportions of subclones also exhibited diversity, and the dominant genotype was also distinguishable between samples. Thus, the evolutionary models of CCLM were complex and the subclones in primary lesions and multiple metastases were diverse, leading to tumor heterogeneity in a single patient.

### The different functional effects of subclones

**Fig. 5:**
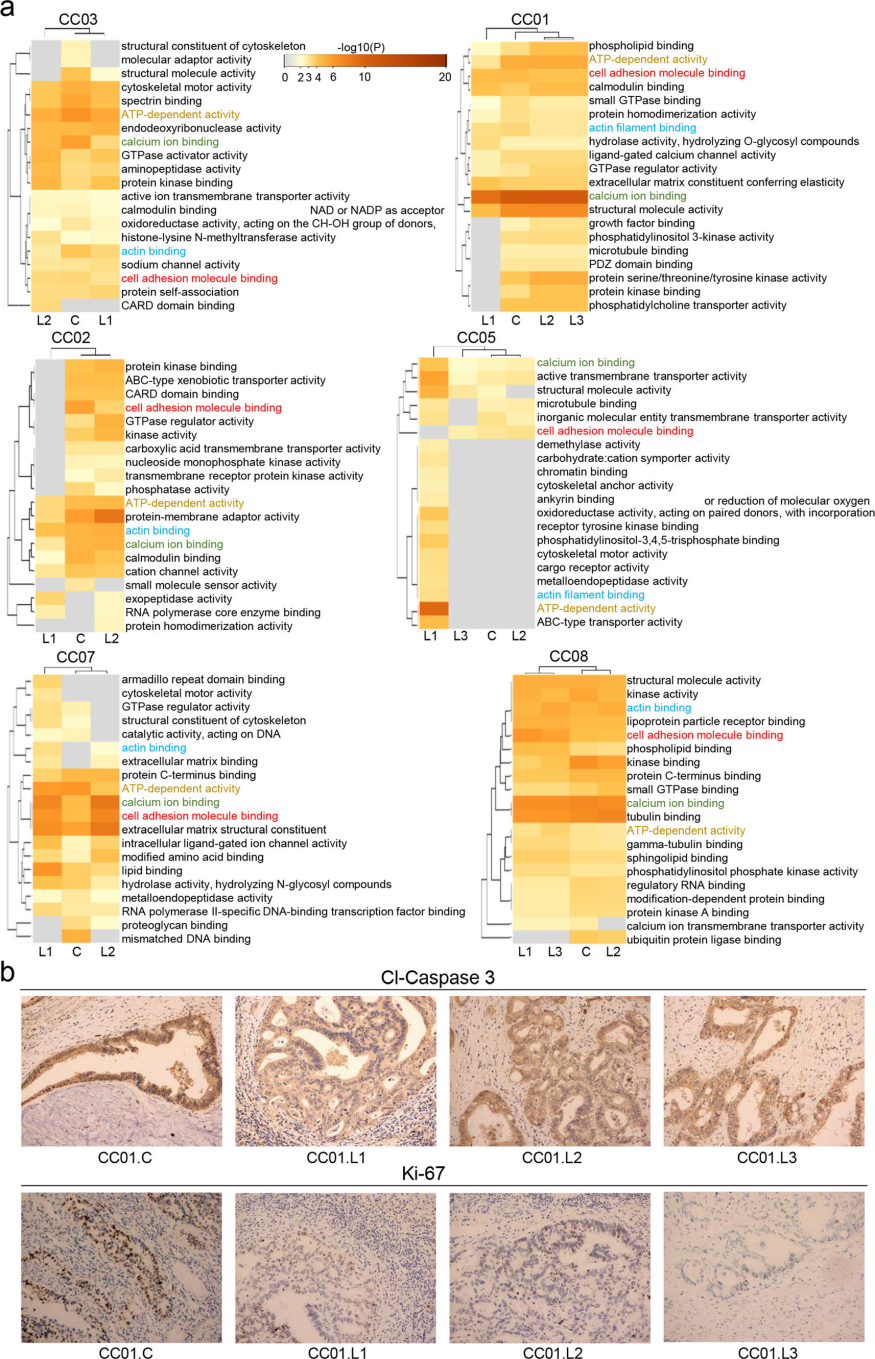
The functional analysis between the primary and metastatic lesions. (**a**) Gene Ontology (GO) was employed to enrich the mutated genes in the involved terms of molecular function, and the significant terms were hierarchically clustered based on Kappa-statistical similarities, among which those with the best p-value were selected and subjected to drawing a heatmap colored by their lg (p-values). Some terms, including ATP-dependent activity, calcium ion binding, actin binding and cell adhesion molecule binding, involved in almost all samples were emphasized in colorful. **b** IHC demonstrated the differential expression of active cleaved-caspase-3 and ki-67 between primary and paired metastatic lesions from CC01, which was observed in ki-67 staining but not in active cleaved-caspase-3 staining

Cancer is a disease of clonal evolution. The selected subclone might lead to cell proliferation, invasion, and metastasis. The subclone in a metastasis might possess the ability to induce different features compared with the genotype of the primary lesion. Hence, pathway and process enrichment analysis was carried out for six patient with different subclones in primary and metastatic lesions using Metascape (Fig. 5a) [23]. As illustrated in the heatmap, several enriched pathways were frequently observed in primary lesions and metastases from all eight patients. These included ATP-dependent activity, calcium ion binding, actin binding, and cell adhesion molecule binding; the latter was observed at almost all sites in these eight patients. In addition, immunohistochemically (IHC) analysis of cell proliferation and apoptosis was performed to further examine biological differences between primary and metastatic lesions in the same case (Fig. 5b, Table 2, Fig. S3, S4). According to IHC staining for cleaved-caspase-3, similar results were obtained for the primary lesions and metastases. However, in case CC03, the staining intensity for the primary lesion was weak and the positive area was 50%. For CC03.L1, the staining intensity was weak and the positive area was 80%. For CC03.L2, the staining intensity was moderate and the positive area was > 90%. Compared with CASP3, Ki-67 exhibit broader heterogeneity in cases CC01, CC05, CC08, CC02, CC03, and CC07.

**Table 2:** Expression of Caspase-3 and Ki-67 in primary and metastasis tissues by the immunofluorenscence staining

	Caspase-3		Ki-67	
	Intensity	Positive area (%)	Intensity	Positive area
CC01.C	++	>90	+	20% (hot spot 50%)
CC01.L1	++	>90	+	30%
CC01.L2	++	>90	+	5%
CC01.L3	++	>90	++	5% (hot spot 50%)
CC02.C	++ ~ +	++90, +10	+	3%
CC02.L1	++	>90	+	10%
CC02.L2	++	>90	+	<1%
CC03.C	+	50	+++ ~ ++	3%
CC03.L1	+	80	+++ ~++	5% (hot spot 60%)
CC03.L2	++	>90	+++	10% (hot spot 60%)
CC05.C	++	90	++	10% (hot spot 50%)
CC05.L1	++	90	++	20% (hot spot 50%)
CC05.L2	+	90	+	5% (hot spot 50%)
CC05.L3	+	90	+++ ~++	10% (hot spot 80%)
CC07.C	+	90	++ ~ +	20% (hot spot 50%)
CC07.L1	++	90	++ ~ +	30% (hot spot 50%)
CC07.L2	++	90	++ ~ +	10% (hot spot 50%)
CC08.C	++	90	+++ ~++	20% (hot spot 60%)
CC08.L1	++	90	+	3% (hot spot 50%)
CC08.L2	++	90	++	5% (hot spot 15%)
CC08.L3	++	90	++	10% (hot spot 60%)

Immunohistochemistry analysis showed the distinction of primary and metastasis tissues, the staining color was scored as light-yellow particle (+), brown-yellow particle (++), and brown particle (+++).

## Discussion

CCLM is a heterogeneous disease with similar phenotype, but different genotypes, prognoses, and responses to chemotherapy. Current tumor treatment strategies are based on the linear evolution model, so if evolution models were diverse, then the treatment strategy should subsequently alter. Nevertheless, few studies have investigated the evolutionary processes responsible for these phenomena. Based on our data, tumor heterogeneity from matched primary lesions and multiple liver metastases of treatment-naïve patients was assessed, all samples displayed intra- and inter-tumor heterogeneity considerably, and the cancer phylogeny tree was also inferred, which implied different evolution models and metastatic route from primary tumor to liver in accord with primary-seeding or metastasis-seeding metastasis models.

It is crucial to understand the driver mutation genes relationship between primary lesions and metastases. Nevertheless, the overlap appears to be more ubiquitous than previously thought, especially in the mostly frequently mutation genes. As demonstrated in Fig. 2b, there were a large number of overlaps in driver gene mutations between primary and metastatic lesions, indicating the top 50 genes in Fig. 2b were mainly homogeneity. This is because, gene mutation empowers the selective advantages of tumor cell, constituting to the regulation of the major cellular functions including growth, death and genome stability and driving tumorigenesis. These mutations with the highest frequency can be inherited to the metastatic lesions without loss throughout the evolution, leading to the overlap in the Fig. 2b. We called these overlapped mutation as “trunk mutation” or “base mutation”, contributing to the genetic homogeneity. Moreover, novel mutations might also generate during or after metastasis, which we called “branch mutation”, and empowered heterogeneity between primary and metastatic lesions. The branch mutations usually have a lower mutation frequency since they occur much later than the trunk mutation, but they determine the evolutionary mode and genetic feature of CCLM. However, other situations should be also taken into consideration. Multiple subclones may exist in the primary lesion, which attribute to multiple clonal origins or new subclone generation during tumor growth. Different metastases might originate from different subclones of primary lesion. For example, in case 1, the top 50 mutations in primary lesion were totally inherited to CC01L2 and CC01L3, whereas a portion of them were missing in CC01L1, implying alternative evolutionary branch in CC01L1. Previous study has also proved that the genetic divergence characterizes the mode of evolution across diverse solid tumor types [24]. It is feasible to distinguish tumors driven by strong positive subclonal selection from those evolving neutrally or under weak selection, revealing different modes of evolution both within and between solid tumor types. This quite differed from the current study. They developed a classifier by simulating spatial tumor growth under different evolutionary modes, whereas the current genetic model was derived from tumor heterogeneity in matched primary lesions and multiple liver metastases.

We suggest that the biological differences between primary and metastatic lesions in a single case may be the result of the accumulation of genetic mutations, which are carried by different subclones in the evolutionary process. In our study, the biological differences between primary and metastatic lesions were indeed observed in some cases such as in case 3, which possessed the linear evolution mode. Nevertheless, these biological differences seemed not obvious in most cases. We believe that "clonal evolution" was confined to the genetic variation in metastatic foci but not to the biological feature. As described above, trunk mutations regulate the major cellular functions including growth, death and genome stability in both primary and metastatic lesion, Nevertheless, the branch mutations empower the heterogeneity, as well determine the evolutionary mode and genetic feature of CCLM, which may help explain why the biological differences were not such obvious.

Currently, drug therapies for CCLM have limited efficacy, thus, targeting the most frequently enriched pathways in metastatic subclones introduced by evolution may be possible to improve the efficacy. Several studies have demonstrated the evolution models in the development of cancer, but few studies have been performed using treatment-naïve samples of CCLM, due to the difficulty in collecting samples from patients. In this case, our research attempted to examine several aspects of treatment-naïve multiple synchronous CCLM, including the functional effects of subclones. The cell adhesion molecule binding was frequently observed in subclones from all samples in our data (Fig. 5), moreover, studies have shown that monoclonal antibodies involved in cell-to-cell regulation of adhesion have been extensively studied as a promising treatment for gastrointestinal cancer [25, 26], as such, it may be a potential treatment for CCLM, but its causal role should be clarified in future investigations. Moreover, mismatch repair deficient and TMB are the crucial features for cancer because of the immune checkpoint inhibitors treatment [27, 28]. Therefore, we analyzed the immunologic microenvironments related genes, MMR, TMB and HLA-I homozygosity, which may also be relevant to immune checkpoint blockade [20, 29, 30]. In our study, the MMR and HLA-I status displayed fully coherence, the immunologic microenvironments related genes were not very clearly distinguishable, whereas TMB exhibited heterogeneity in primary lesions and metastases exhibited. Hence, it is preferable to develop a more standardized approach for the treatment of CCLM patients with immune checkpoint inhibitors.

Several limitations of the present study should be acknowledged. Firstly, a major limitation of this study is the lack of matched non-tumor tissue from each patient. Nevertheless, in the absence of control, Mutect2 can use a single sample as input; also, a pooled standard sample can be used as normal. Mutect, a method developed by Broad Institute (Cambridge, MA, USA) for the reliable and accurate identification of somatic point mutations using NGS data, has been applied successively identify the sites carrying somatic mutations with high confidence. Secondly, we performed only whole-exome sequencing without RNA sequencing to investigate the evolution of the tumor immune microenvironment.

Collectively, our research demonstrated the genetic and functional similarities and differences between primary and metastatic lesions, as well as among multiple metastases, based on which three natural history models of CCLM were identified including parallel, linear, and branching, revealing liver metastasis originated not only from the primary but also from another metastatic lesion. Our results provide the genomic evidence for the metastatic heterogeneity and evolution of CCLM, which should be considered for its therapeutic decision making in future.

## Supplementary Information

Below is the link to the electronic supplementary material.

Supplementary file1 (PDF 619 KB)

Supplementary file2 Comparison of somatic mutations between primary and metastases. (a) Comparison of TMB between primary and metastases. (b) The rest eight gene and KRAS, PIK3CA lollipop mutation diagrams of primary (pointing up) and metastases (pointing down). Different color patches represent different domains, and y-axes means the number of mutations in primary and metastases, note that multiple mutation in a gene may occur from some case.(JPG 1245 KB)

Supplementary file3 The immune-associated gene profile of primary and metastases. The mutations were picked out based on the immune-associated genes including antigen processing and presentation, antimicrobials, BCR signaling, pathway, chemokine receptors, chemokines, cytokine receptors, cytokines, natural killer cell cytotoxicity, TCR signaling pathway. (JPG 1131 KB)

Supplementary file4 Intra and inter-tumor heterogeneity and subclones functional analysis. (a-b) The rest seven cases intra- and inter-tumor heterogeneity plots. The violin plots are depicted at the left, and cellular prevalence of each cluster across the different samples are at right. (JPG 1863 KB)

Supplementary file5 (JPG 1958 KB)

Supplementary file6 Immunohistochemical analysis showed expression of active cleaved-caspase-3 of primary and paired metastases of CC05, CC08, CC02, CC03, CC07. (JPG 2367 KB)

Supplementary file7 Immunohistochemical analysis showed expression of active ki-67 of primary and paired metastases of CC05, CC08, CC02, CC03, CC07. (JPG 2111 KB)

Supplementary file8 (DOCX 18 kb)

## References

[1] Bray F, Ferlay J, Soerjomataram I (2018). Global cancer statistics 2018: GLOBOCAN estimates of incidence and mortality worldwide for 36 cancers in 185 countries. CA Cancer J Clin.

[2] Teng S, Li YE, Yang M (2020). Tissue-specific transcription reprogramming promotes liver metastasis of colorectal cancer. Cell Res.

[3] Dang HX, Krasnick BA, White BS, et al. The clonal evolution of metastatic colorectal cancer. Sci Adv. 2020; 6 eaay9691. 10.1126/sciadv.aay9691 PMC7286679 32577507

[4] Greaves M, Maley CC (2012). Clonal evolution in cancer. Nature.

[5] Merlo LM, Pepper JW, Reid BJ (2006). Cancer as an evolutionary and ecological process. Nat Rev Cancer.

[6] Grady WM, Carethers JM (2008). Genomic and epigenetic instability in colorectal cancer pathogenesis. Gastroenterology.

[7] Fearon ER, Vogelstein B (1990). A genetic model for colorectal tumorigenesis. Cell.

[8] Gerlinger M, Rowan AJ, Horswell S (2012). Intratumor heterogeneity and branched evolution revealed by multiregion sequencing. N Engl J Med.

[9] Bozic I, Reiter JG, Allen B (2013). Evolutionary dynamics of cancer in response to targeted combination therapy. Elife.

[10] Yang J, Lin Y, Huang Y (2019). Genome landscapes of rectal cancer before and after preoperative chemoradiotherapy. Theranostics.

[11] Fan J, Lee HO, Lee S (2018). Linking transcriptional and genetic tumor heterogeneity through allele analysis of single-cell RNA-seq data. Genome Res.

[12] Seretis F, Seretis C, Youssef H (2014). Colorectal cancer: seed and soil hypothesis revisited. Anticancer Res.

[13] Langley RR, Fidler IJ (2011). The seed and soil hypothesis revisited–the role of tumor-stroma interactions in metastasis to different organs. Int J Cancer.

[14] Paget S. The distribution of secondary growths in cancer of the breast. 1889. Cancer Metastasis Rev. 1989; 8:98–101. 2673568

[15] Wang K, Li M, Hakonarson H (2010). ANNOVAR: functional annotation of genetic variants from high-throughput sequencing data. Nucleic Acids Res.

[16] Bambury RM, Bhatt AS, Riester M (2015). DNA copy number analysis of metastatic urothelial carcinoma with comparison to primary tumors. BMC Cancer.

[17] Talevich E, Shain AH, Botton T (2016). CNVkit: Genome-wide copy number detection and visualization from targeted DNA sequencing. PLoS Comput Biol.

[18] Cao J, Chen L, Li H (2019). An accurate and comprehensive clinical sequencing assay for cancer targeted and immunotherapies. Oncologist.

[19] Chalmers ZR, Connelly CF, Fabrizio D (2017). Analysis of 100,000 human cancer genomes reveals the landscape of tumor mutational burden. Genome Med.

[20] Chowell D, Morris LGT, Grigg CM (2018). Patient HLA class I genotype influences cancer response to checkpoint blockade immunotherapy. Science.

[21] Roth A, Khattra J, Yap D (2014). PyClone: statistical inference of clonal population structure in cancer. Nat Methods.

[22] Popic V, Salari R, Hajirasouliha I (2015). Fast and scalable inference of multi-sample cancer lineages. Genome Biol.

[23] Cao CH, Liu R, Lin XR (2021). LRP1B mutation is associated with tumor HPV status and promotes poor disease outcomes with a higher mutation count in HPV-related cervical carcinoma and head & neck squamous cell carcinoma. Int J Biol Sci.

[24] Sun R, Hu Z, Sottoriva A (2017). Between-region genetic divergence reflects the mode and tempo of tumor evolution. Nat Genet.

[25] Sagiv E, Arber N (2008). The novel oncogene CD24 and its arising role in the carcinogenesis of the GI tract: from research to therapy. Expert Rev Gastroenterol Hepatol.

[26] Eyvazi S, Kazemi B, Dastmalchi S (2018). Involvement of CD24 in multiple cancer related pathways makes it an interesting new target for cancer therapy. Curr Cancer Drug Targets.

[27] Diaz LA, Shiu KK, Kim TW (2022). Pembrolizumab versus chemotherapy for microsatellite instability-high or mismatch repair-deficient metastatic colorectal cancer (KEYNOTE-177): final analysis of a randomised, open-label, phase 3 study. Lancet Oncol.

[28] Shimada Y, Okuda S, Watanabe Y (2021). Histopathological characteristics and artificial intelligence for predicting tumor mutational burden-high colorectal cancer. J Gastroenterol.

[29] Chowell D, Krishna C, Pierini F (2019). Evolutionary divergence of HLA class I genotype impacts efficacy of cancer immunotherapy. Nat Med.

[30] Abed A, Calapre L, Lo J (2020). Prognostic value of HLA-I homozygosity in patients with non-small cell lung cancer treated with single agent immunotherapy. J Immunother Cancer.

[31] Chen T, Chen X, Zhang S (2021). The genome sequence archive family: toward explosive data growth and diverse data types. Genomics Proteomics Bioinformatics.

[32] Members C-N, Partners. Database Resources of the National Genomics Data Center, China National Center for Bioinformation in 2022. Nucleic Acids Res. 2022; 50:D27-D38. 10.1093/nar/gkab951 PMC8728233 34718731

