# Supplementary material for: The clonal heterogeneity of colon cancer with liver metastases
Source: J Gastroenterol. 2023 Apr 12;58(7):642–55. doi: 10.1007/s00535-023-01989-6 (PMC10307713; doi:10.1007/s00535-023-01989-6)
Supplement: Supplementary file 1 — Supplementary file1 (PDF 619 KB) [file 535_2023_1989_MOESM1_ESM.pdf]

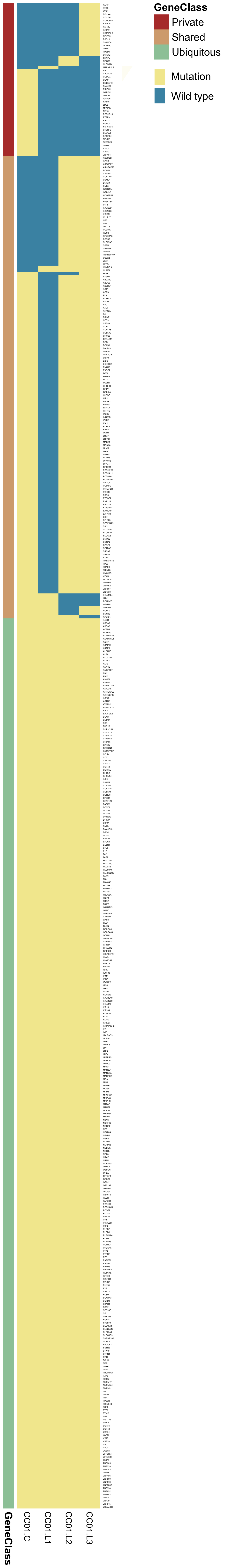
GeneClass
Private
Shared
Ubiquitous
Mutation
Wild type
GeneClass
CC01.C
CC01.L1
CC01.L2
CC01.L3

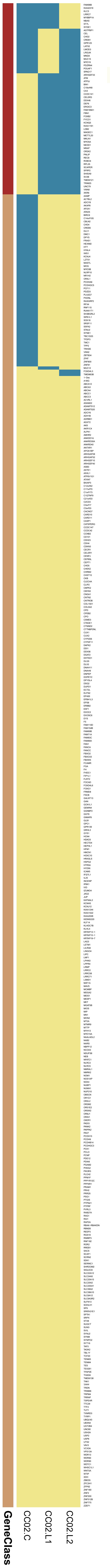
GeneClass
CO2.C
CO2.L1
CO2.L2

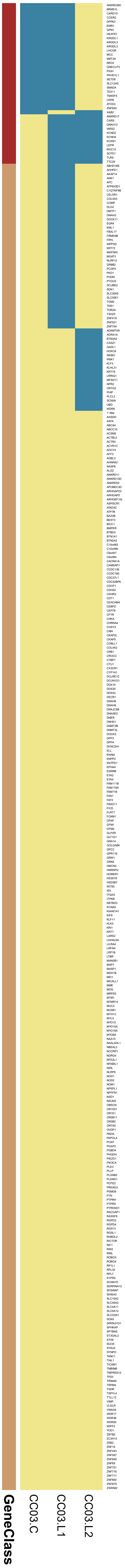
GeneClass
CC03.C
CC03.L1
CC03.L2

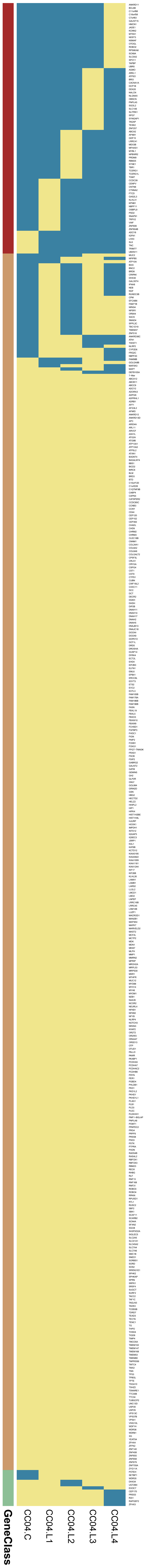
GeneClass
CC04.C
CC04.L1
CC04.L2
CC04.L3
CC04.L4

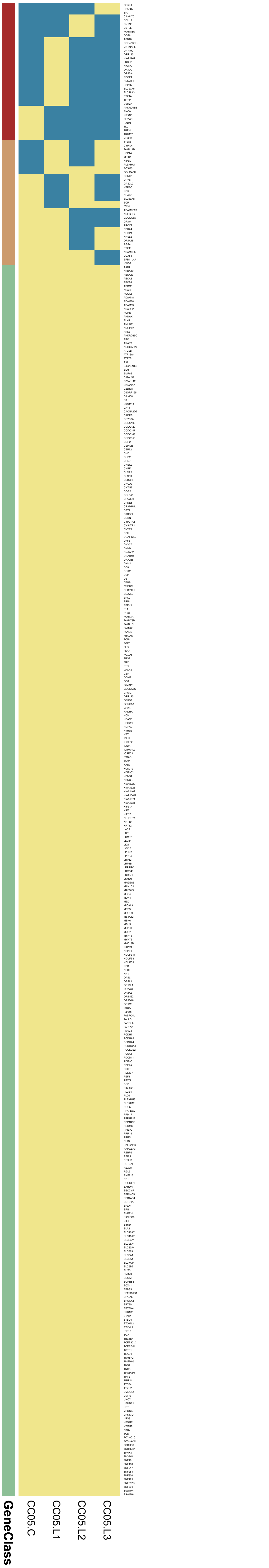
GeneClass
CC05.C
CC05.L1
CC05.L2
CC05.L3

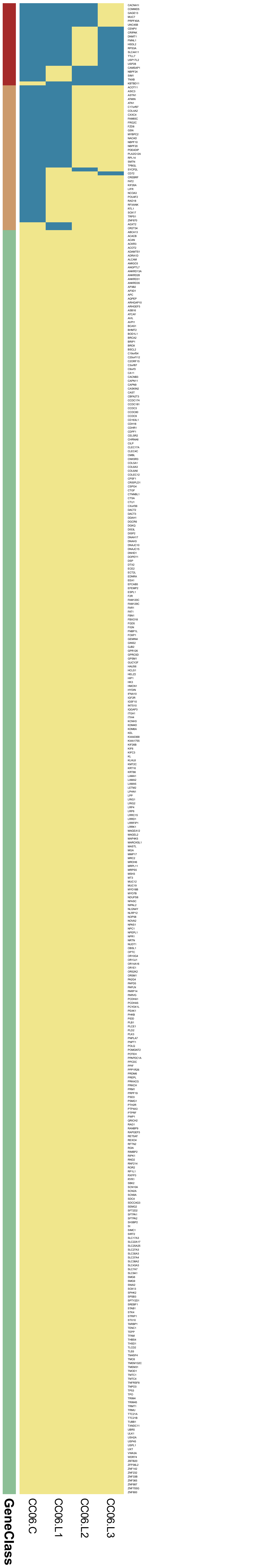
GeneClass
CC06.C
CC06.L1
CC06.L2
CC06.L3

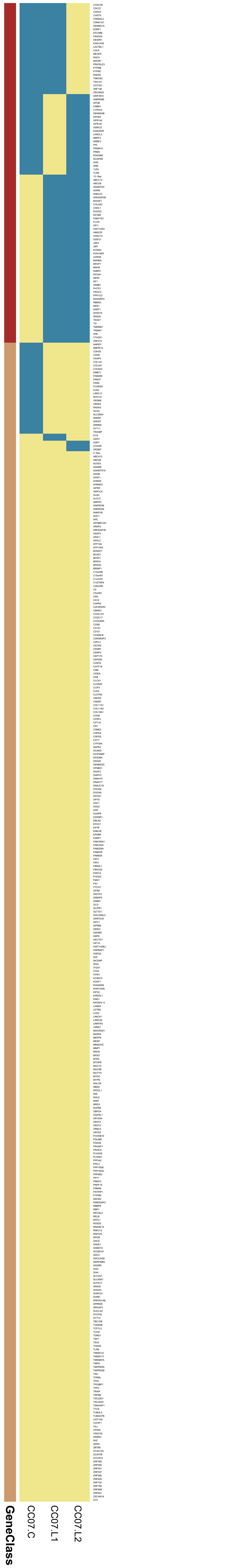
GeneClass
CC07.C
CC07.L1
CC07.L2

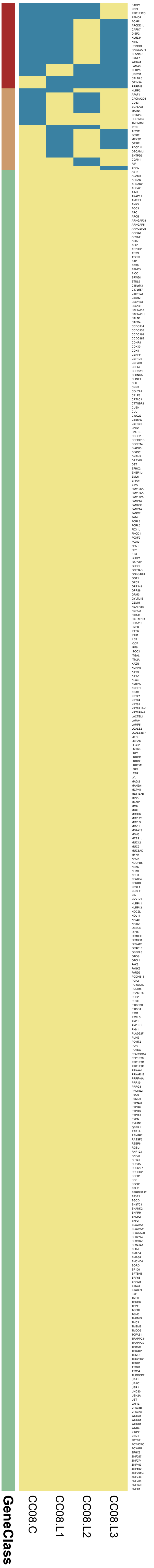
GeneClass
CC08.C
CC08.L1
CC08.L2
CC08.L3
