# Supplementary figures and images for: The clonal heterogeneity of colon cancer with liver metastases
Source: J Gastroenterol. 2023 Apr 12;58(7):642–55. doi: 10.1007/s00535-023-01989-6 (PMC10307713; doi:10.1007/s00535-023-01989-6)

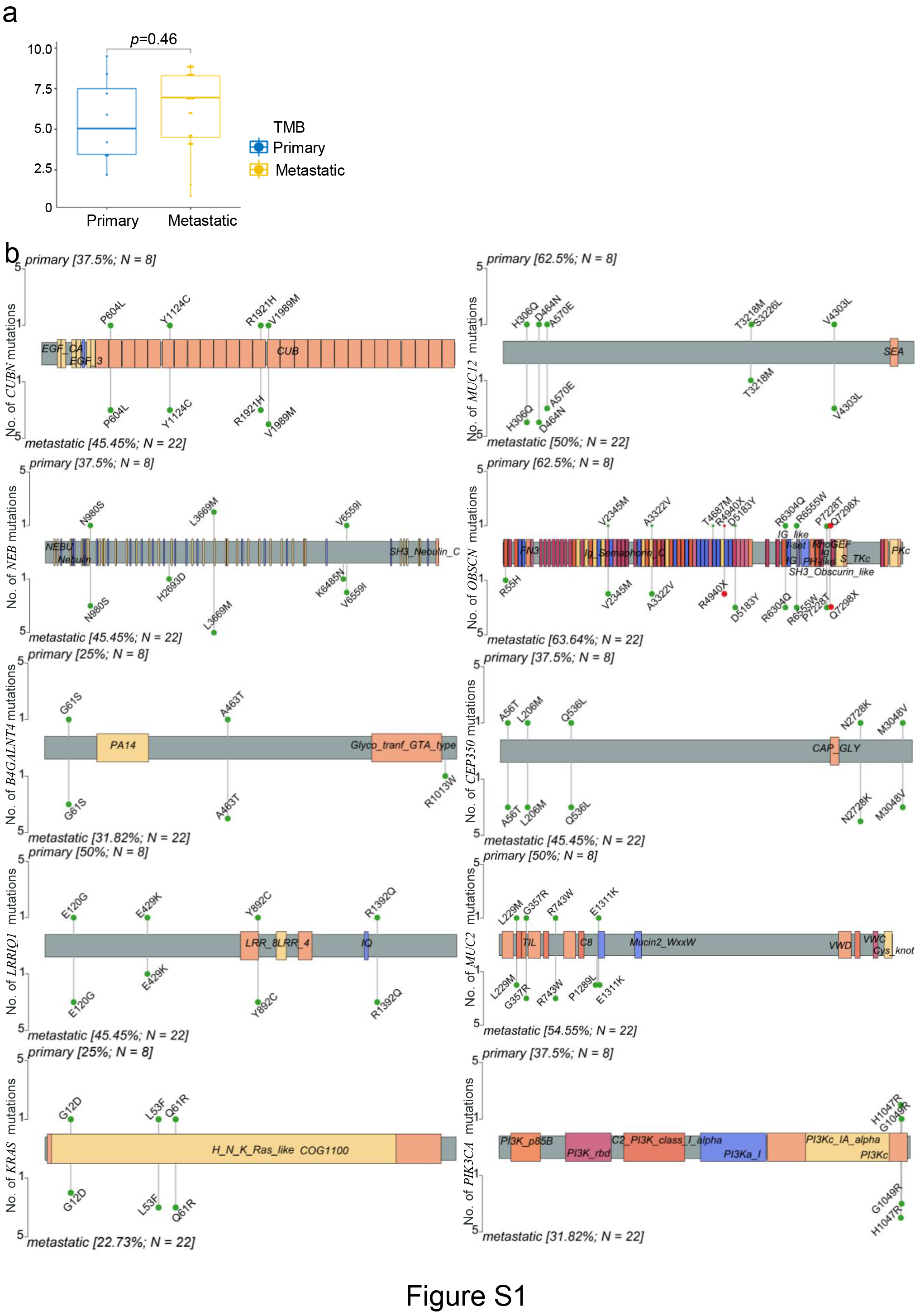

Supplement: Supplementary file 2 — Supplementary file2 Comparison of somatic mutations between primary and metastases. (a) Comparison of TMB between primary and metastases. (b) The rest eight gene and KRAS, PIK3CA lollipop mutation diagrams of primary (pointing up) and metastases (pointing down). Different color patches represent different domains, and y-axes means the number of mutations in primary and metastases, note that multiple mutation in a gene may occur from some case.(JPG 1245 KB) [file 535_2023_1989_MOESM2_ESM.jpg]

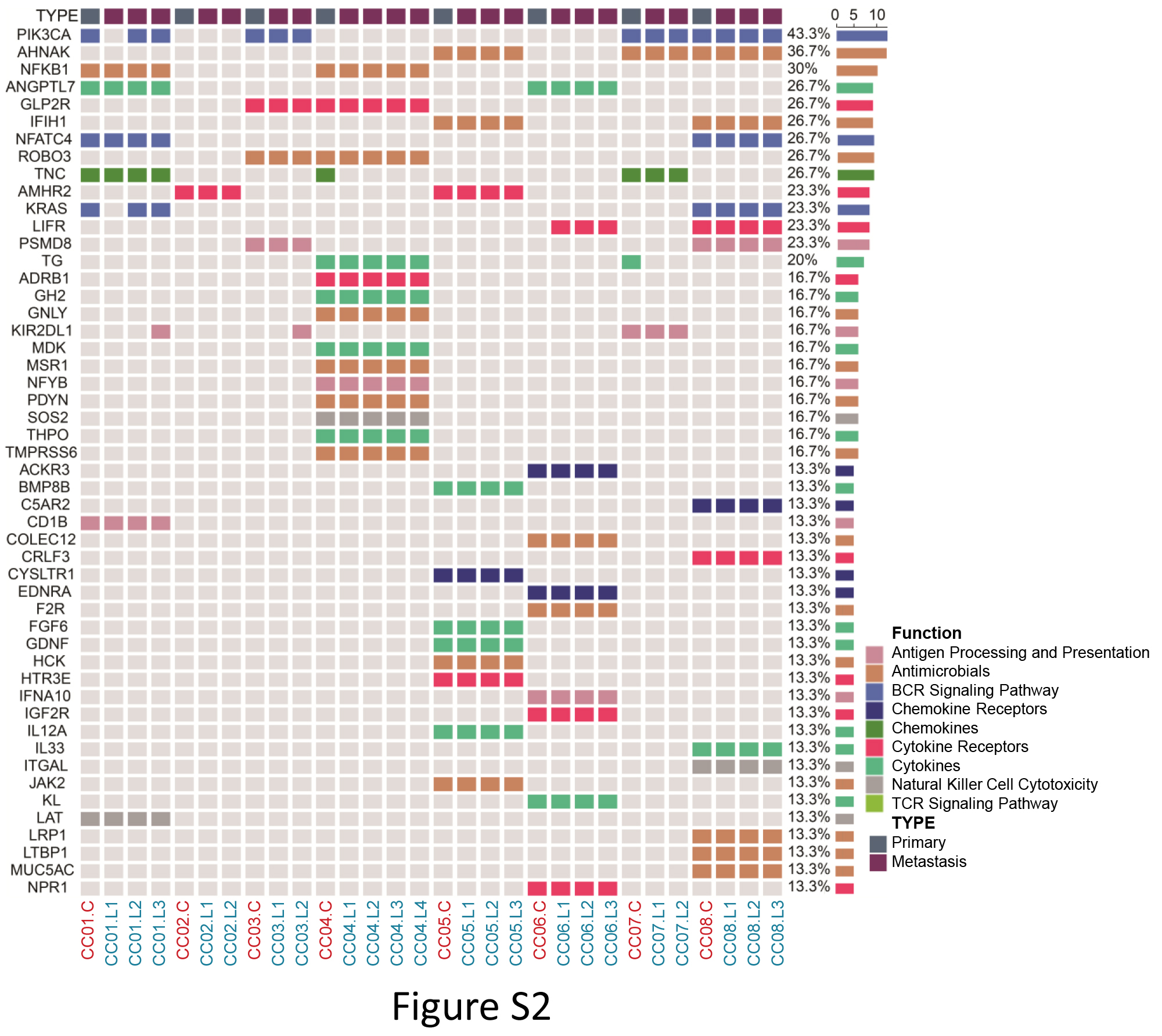

Supplement: Supplementary file 3 — Supplementary file3 The immune-associated gene profile of primary and metastases. The mutations were picked out based on the immune-associated genes including antigen processing and presentation, antimicrobials, BCR signaling, pathway, chemokine receptors, chemokines, cytokine receptors, cytokines, natural killer cell cytotoxicity, TCR signaling pathway. (JPG 1131 KB) [file 535_2023_1989_MOESM3_ESM.jpg]

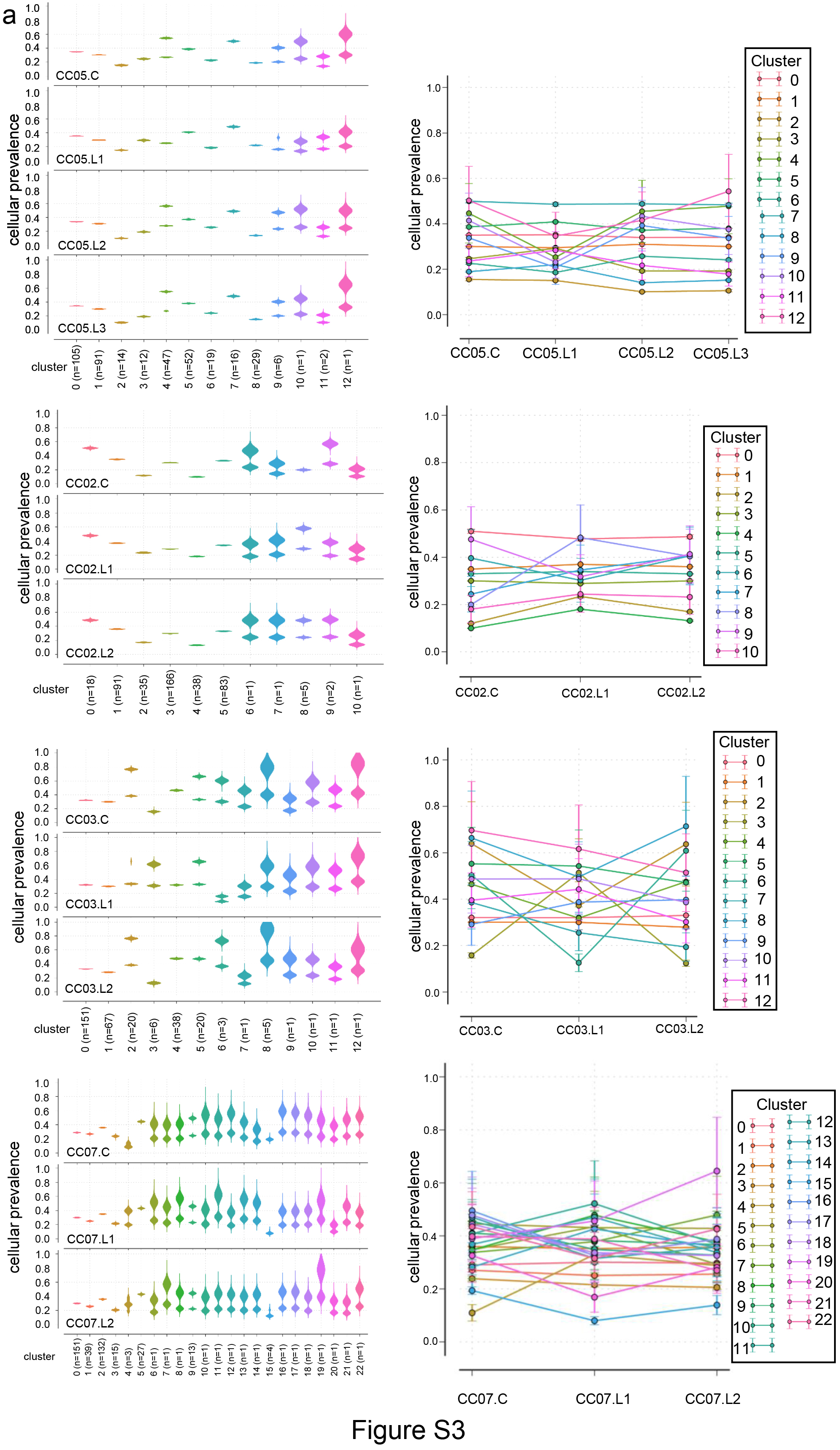

Supplement: Supplementary file 4 — Supplementary file4 Intra and inter-tumor heterogeneity and subclones functional analysis. (a-b) The rest seven cases intra- and inter-tumor heterogeneity plots. The violin plots are depicted at the left, and cellular prevalence of each cluster across the different samples are at right. (JPG 1863 KB) [file 535_2023_1989_MOESM4_ESM.jpg]

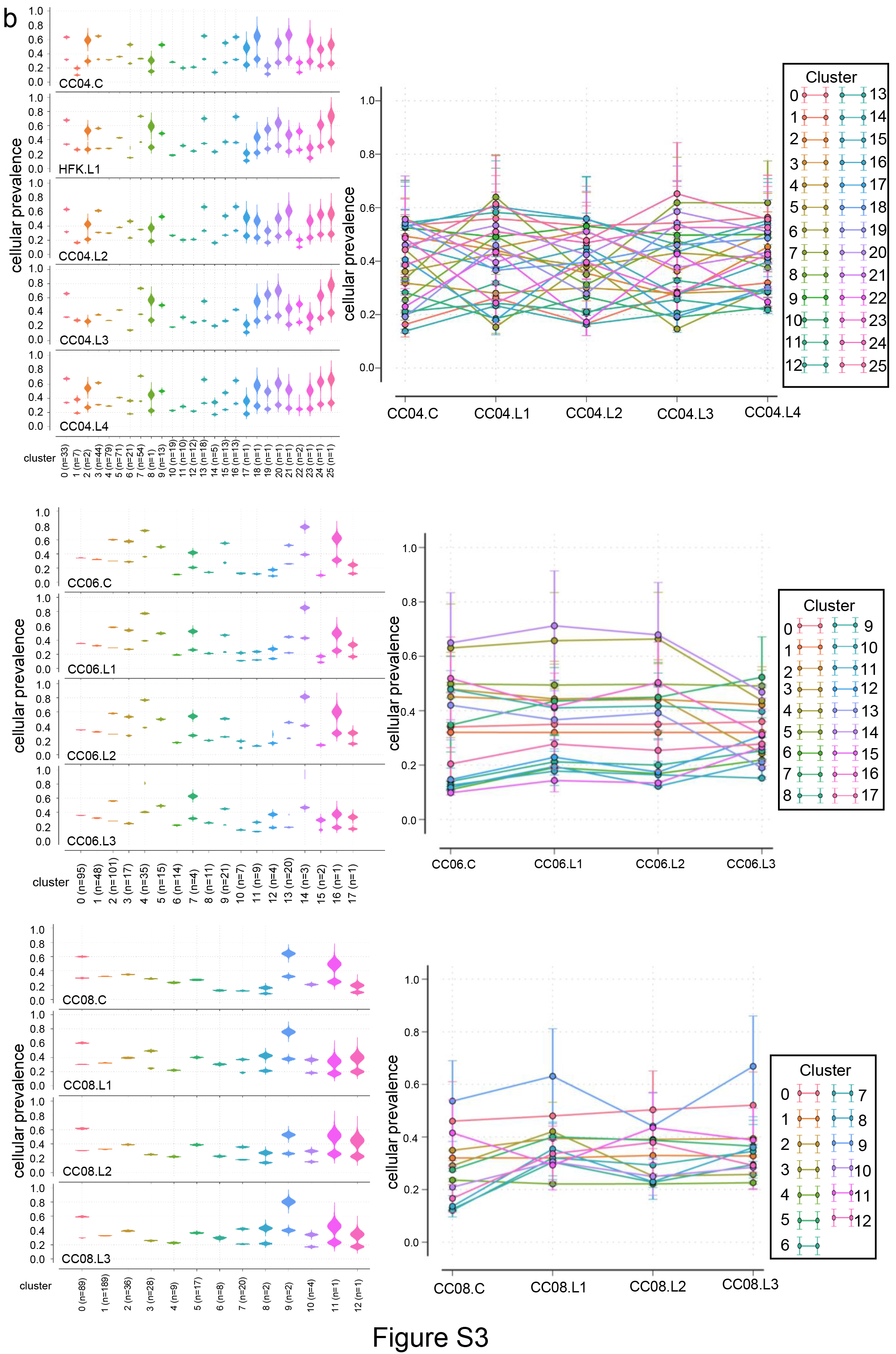

Supplement: Supplementary file 5 — Supplementary file5 (JPG 1958 KB) [file 535_2023_1989_MOESM5_ESM.jpg]

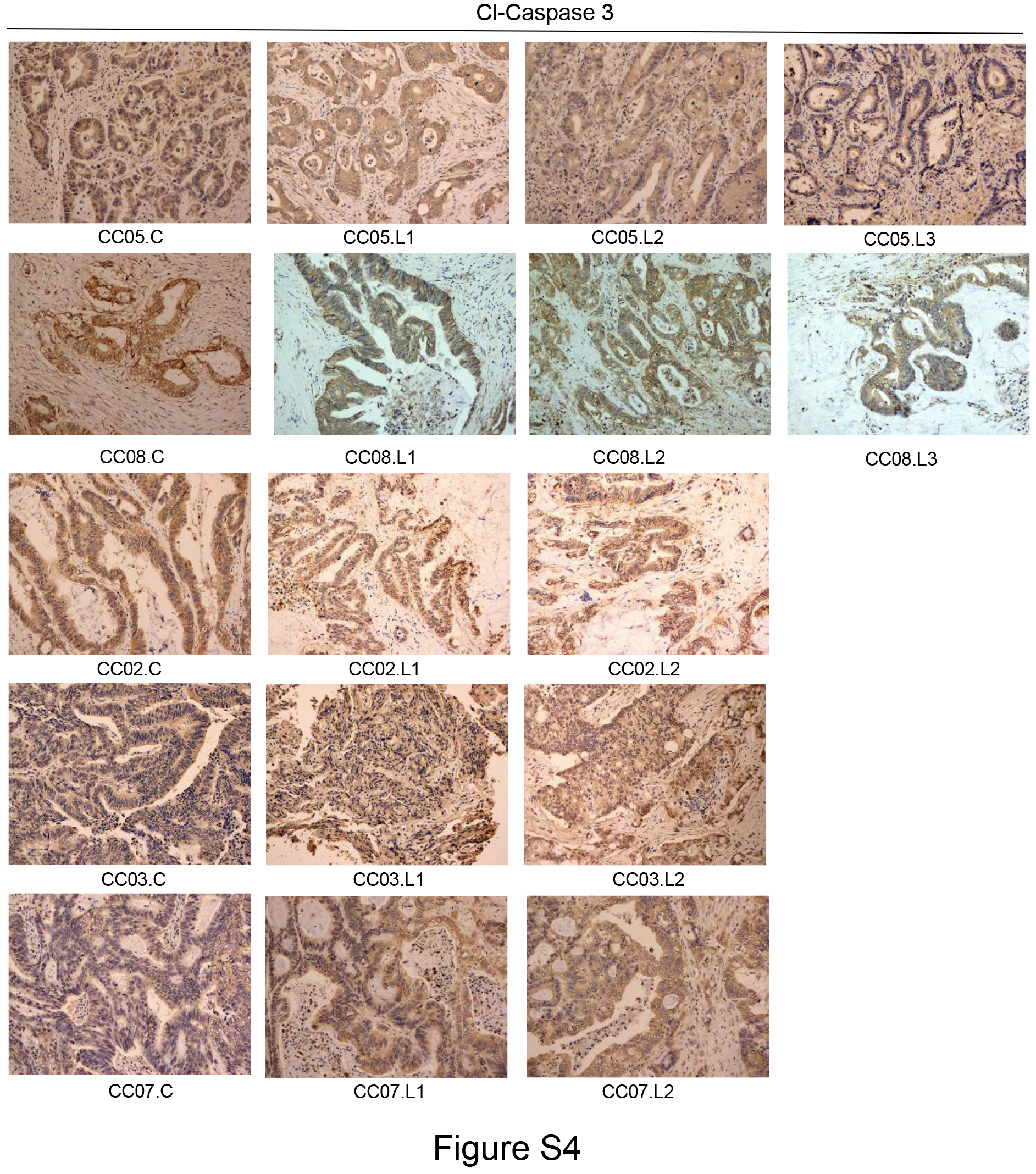

Supplement: Supplementary file 6 — Supplementary file6 Immunohistochemical analysis showed expression of active cleaved-caspase-3 of primary and paired metastases of CC05, CC08, CC02, CC03, CC07. (JPG 2367 KB) [file 535_2023_1989_MOESM6_ESM.jpg]

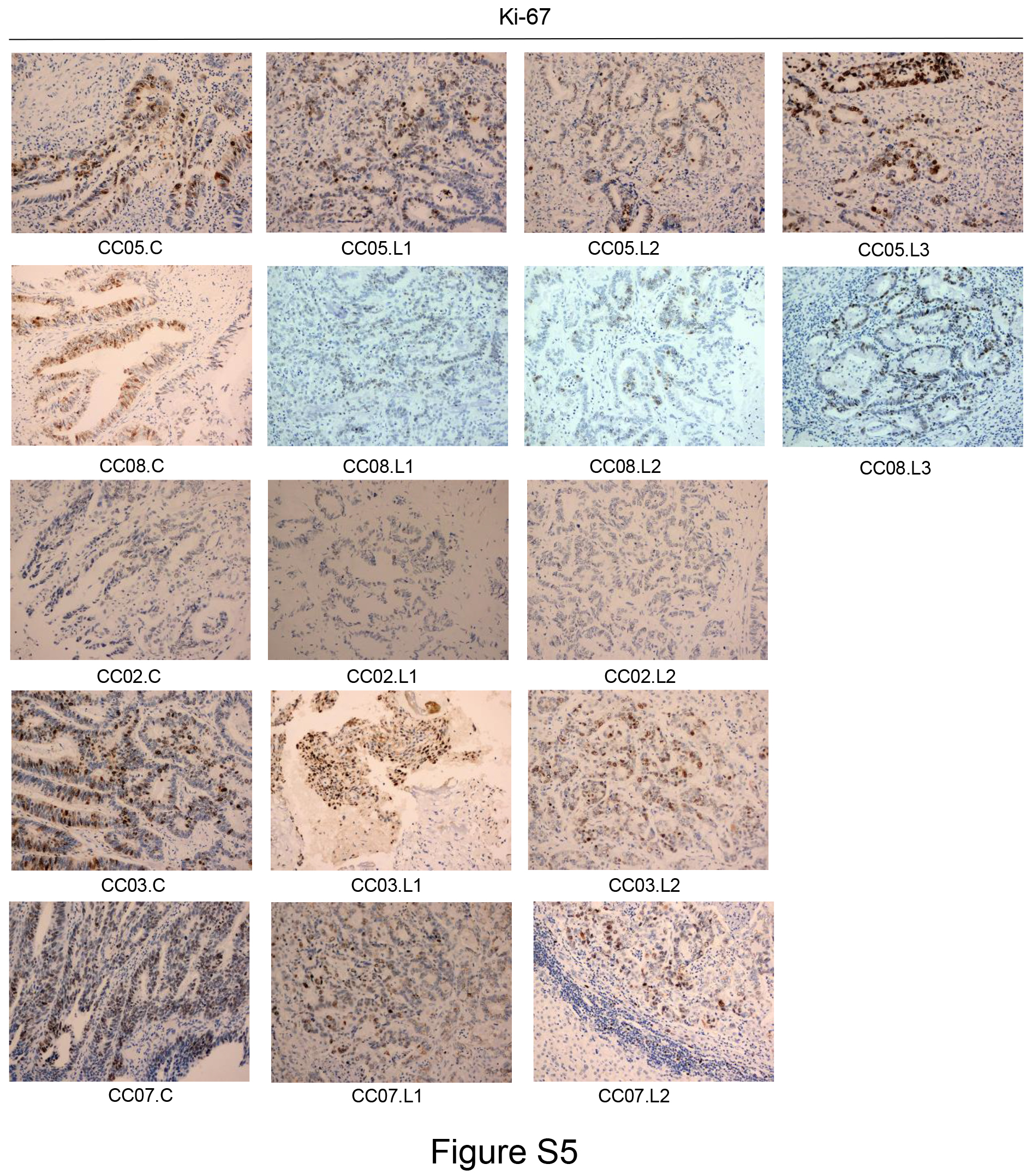

Supplement: Supplementary file 7 — Supplementary file7 Immunohistochemical analysis showed expression of active ki-67 of primary and paired metastases of CC05, CC08, CC02, CC03, CC07. (JPG 2111 KB) [file 535_2023_1989_MOESM7_ESM.jpg]
