# Supplementary material for: The clonal heterogeneity of colon cancer with liver metastases
Source: J Gastroenterol. 2023 Apr 12;58(7):642–55. doi: 10.1007/s00535-023-01989-6 (PMC10307713; doi:10.1007/s00535-023-01989-6)
Supplement: Supplementary file 8 — Supplementary file8 (DOCX 18 kb) [file 535_2023_1989_MOESM8_ESM.docx]

Table S1. the mutation information of NRAS and BRAF

|  | mutation site | |
| --- | --- | --- |
| sample name | PIK3CA | KRAS |
| CC01.C | K111_I112delinsN | G12D |
| CC01.L1 | NA | NA |
| CC01.L2 | K111_I112delinsN | G12D |
| CC01.L3 | K111_I112delinsN | G12D |
| CC03.C | H1047R | NA |
| CC03.L1 | H1047R | NA |
| CC03.L2 | H1047R | NA |
| CC07.C | H1047R | NA |
| CC07.L1 | H1047R | NA |
| CC07.L2 | H1047R | NA |
| CC08.C | H1047R | Q61R and L53F |
| CC08.L1 | H1047R | Q61R and L53F |
| CC08.L2 | H1047R | Q61R and L54F |
| CC08.L3 | H1047R | Q61R and L55F |
